# Unraveling the Complexities of Silica Nanoparticle Adsorption onto Polymer Latexes in Pickering Emulsion Polymerization

**DOI:** 10.1021/acs.langmuir.4c02219

**Published:** 2024-08-19

**Authors:** Zekai Shen, Tianheng Wang, Jing Luo, Ren Liu, To Ngai, Guanqing Sun

**Affiliations:** † Key Laboratory of Synthetic and Biological Colloids, Ministry of Education, School of Chemical and Material Engineering, Jiangnan University, Wuxi, 214122 Jiangsu, China; ‡ Department of Chemistry, The Chinese University of Hong Kong, Shatin N.T., Hong Kong, 999077 Special Administrative Region, China

## Abstract

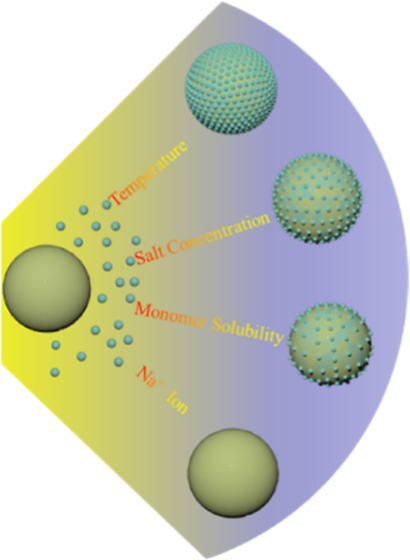

Incorporating unmodified silica nanoparticles onto polymer latexes to fabricate aqueous polymer dispersions without relying on electrostatic attraction during the Pickering emulsion polymerization process still faces challenges. For negatively charged silica nanoparticles to successfully adsorb onto polymer latexes, particularly in an anionic initiator emulsion polymerization system, they have remained elusive without the use of auxiliary monomers and cationic initiators. This study investigates various experimental parameters, such as emulsion polymerization temperature, monomer solubility, salt concentration, and cation type, to elucidate the factors influencing the adsorption of unmodified silica nanoparticles in Pickering emulsion polymerization. While poly(methyl methacrylate) (PMMA)/SiO_2_ hybrid latexes can be obtained under pH conditions of 5–6 and at temperatures of 65 °C or below, the loading rate of silica nanoparticles decreases as the reaction temperature increases, resulting in bare PMMA latexes without silica nanoparticle adsorption at temperatures exceeding 70 °C. Introducing styrene (St) into the monomer mixture with methyl methacrylate in a ratio of up to 10 wt % leads to a gradual decrease in silica nanoparticle loading rate, from 27.3 to 8.2 wt %, attributed to the low solubility of St in water. Furthermore, the presence of sodium ions (Na^+^) is found to be crucial for silica nanoparticle adsorption onto PMMA latexes, as the sodium ions have a stabilizing effect on both the silica nanoparticles and the silica nanoparticle-armored latexes. These findings highlight the complex nature of Pickering emulsion polymerization in the presence of unmodified silica nanoparticles, demonstrating that the loading rate of silica nanoparticles onto polymer latexes is influenced by various factors. These insights pave the way for developing aqueous polymer dispersions with high silica nanoparticle loading rates onto polymer latexes, which is a desirable trait in the coating industry.

## Introduction

The adsorption of solid nanoparticles onto the oil–water interface has been a subject of investigation for over a century, starting with the groundbreaking work of Ramsden and Pickering et al.^1−4^ In the past two decades, extensive research studies on emulsions and foams, focusing on stabilization mechanisms and potential applications across various fields, have been conducted by both academic and industrial researchers.^5,6^ It is now well established that the adsorption of solid nanoparticles onto the oil–water interface occurs due to the reduction of oil–water interfacial energy, making the adsorption process energetically favorable, as indicated by thermodynamic calculations.^7^ Various solid nanoparticles, including silica nanoparticles, Laponite nanoplates, magnetic nanoparticles, titania nanoparticles, and graphene oxide nanoplates, have been utilized to stabilize oil–water interfaces.^8^ Through solidification of the oil phase if polymerizable oil is used, hybrid polymer particles with inorganic particles adsorbed on the surface can be produced.^9^ However, these polymer particles armored with inorganic particles replicate the original emulsion droplets, and the polymerization mechanism is basically suspension polymerization, resulting in large particle sizes and broad size distributions, which impedes their use as film-forming materials in applications such as coatings, inks, and adhesives.^10^

To prepare synthetic polymer latexes dispersed in water, small molecular surfactants are an indispensable ingredient in traditional emulsion polymerization. Typically, 1.0–5.0 wt % surfactants are present in the original emulsion latex, and their concentration is increased with evaporation of water. Consequently, surfactants are undesirable in coating, ink, and adhesive end applications.^11,12^ The surfactants, mostly small molecules, can damage the water resistance of cured films by constructing water transportation channels inside the film which seriously deteriorates the anticorrosion performance of the waterborne latex coating.^13−15^ As an alternative stabilizer of small molecular and polymeric surfactants, solid particles such as carbon black actually have been used in the early development of emulsion polymerization.^16,17^ However, the role of solid particles in emulsion polymerization is much less investigated and discussed compared with emulsion polymerization in the presence of surfactants. It is highly appealing to coating, adhesive, and ink industries that solid particle-stabilized emulsion latexes can be prepared in the absence of surfactants.^18^ In the past two or three decades, there has been growing research effort in academia to conduct emulsion polymerization in the presence of fine solid nanoparticles rather than small molecular surfactants. Until now, a variety of solid particles, such as silica particles,^19^ Laponite clay,^20^ magnetic particles,^21^ titanium dioxide particles,^22^ graphene oxide sheets,^23^ and polymer nanogels,^24^ have been successfully used to prepare hybrid latexes through Pickering emulsion polymerization. Of them, silica nanoparticles are of special interest due to their industrial availability and cheap price. Percy et al. and others reported in 1999 the free radical emulsion polymerization in the presence of silica nanoparticles in an aqueous phase with 4-vinylpyridine as the cationic comonomer. They found that silica nanoparticles with negative surface charge can show strong acid–base interactions with the 4-vinylpyridine-functionalized latexes, leading to silica-armored hybrid poly(methyl methacrylate) (PMMA) and polystyrene (PSt) latexes with the help of electrostatic attraction of cationic monomers toward negatively charged radicals. In the absence of silica nanoparticles, the polymerization would lead to naked-eye visible flocculation, clearly demonstrating the stabilizing role of silica nanoparticles in the emulsion polymerization.^25^ Chen and colleagues reported in 2005 the synthesis of PMMA emulsion latexes coated with silica nanoparticles with cationic monomer, 2-(methacryloyl chloride)ethyl trimethylammonium chloride (MTC). This suggests that the electrostatic interaction between negatively charged silica particles and positively charged MTC anchors the silica nanoparticles on the surface of the latexes. They proposed that MTC-modified silica particles act as stabilizers in emulsion polymerization. To some extent, MTC adjusted the wettability of silica particles, promoting their adhesion to the surface of polymer latexes.^26^ Schmid et al. reported that Pickering emulsion in the presence of glycerol-modified silica nanoparticles can produce core–shell latexes armored with silica nanoparticles when 2,2′-azobis(isobutyramidine) dihydrochloride (AIBA) was used as an initiator without the use of auxiliary cationic monomers. The initiator AIBA can be decomposed to generate free radicals with positive charge, and they can be adsorbed onto negatively charged silica nanoparticles which eventually result in the formation of silica nanoparticle-armored latexes.^27^ Therefore, in Pickering emulsion polymerization, the adsorption of silica nanoparticles can be promoted by electrostatic attractions as well as by nonspecific van der Waals forces by selecting suitable monomers/comonomers.

Bon et al. were among the first to conduct Pickering emulsion polymerization using unmodified silica nanoparticles as sole stabilizers for Pickering emulsion polymerization without the use of cationic monomers, surfactants, or cationic initiators.^18^ The study indicated that silica nanoparticle-armored hybrid latexes could only be obtained at pH around 5.5 when using methyl methacrylate (MMA) and ethyl methacrylate as the monomer with hydrophilic silica nanoparticles and anionic initiator potassium persulfate (KPS) as the initiator in the emulsion polymerization system. Using styrene or butyl methacrylate, silica nanoparticles could not adsorb on the surface of polymer latexes, resulting in bare latex particle surfaces, while silica nanoparticles remained extensively in the aqueous phase. It was also shown that silica nanoparticles did not adsorb onto monomer droplets before polymerization.^28^ Therefore, the reaction mechanism and stabilization mechanism of Pickering emulsion polymerization may differ from the stabilization mechanism of the Pickering emulsion. Many experts and scholars have studied the mechanism of Pickering emulsion polymerization. Bon and Lotierzo reported a possible mechanism for Pickering emulsion polymerization in 2017 that initiation of monomers dissolved in water by KPS can generate a low-molecular-weight radical initiator, and subsequent deposition of this oligomer onto silica nanoparticles is the nucleation of armored latexes in Pickering emulsion polymerization.^28^ Ip et al. prepared hybrid latexes via electrostatic interaction between silica nanoparticles and low-molecular-weight radical initiators with opposite charges.^29^ This further demonstrates that electrostatic attraction is crucial in promoting the adsorption of silica nanoparticles onto polymer latexes. However, there are no electrostatic attractions if KPS is used as an initiator in Pickering emulsion polymerization, and there are still no well-established theory and systematic experimental results to elucidate the influencing factors and polymerization mechanism of Pickering emulsion polymerization with unmodified silica nanoparticles without the use of negative and positive charge attraction which is undesirable in emulsion polymerization to promote silica nanoparticle adsorption onto latexes.^30^

In this study, unmodified Ludox TM-40 silica nanoparticles were employed as Pickering stabilizers and KPS as the initiator to conduct Pickering emulsion polymerization in the absence of any auxiliary comonomers, and a series of experiments are designed to elucidate the underlying factors that control the adsorption of silica nanoparticles during Pickering emulsion polymerization. MMA and St are used as representative monomers with high and low solubility in water, respectively. The investigation aimed to explore the effects of polymerization temperature, monomer hydrophobicity, and solubility in water and the crucial role of sodium ions in the morphology of hybrid latexes and the loading rate of silica nanoparticles on polymer latexes.

## Materials and Methods

Potassium persulfate (KPS), styrene (St), and MMA of analytical grade are from Sinopharm Chemical Reagent Co., Ltd., Shanghai, China. St and MMA were purified to remove the inhibitor before use. Sodium chloride, potassium chloride, and sodium sulfate with a purity of >99.0% were purchased from Sinopharm Chemical Reagent Co., Ltd., Shanghai. Ludox TM-40 colloidal silica (40 wt % suspension in H_2_O) is from Sigma-Aldrich. Ultrapure water from a Millipore pure water system is used throughout all the experiments.

## Methods

### Preparation of Latexes via Pickering Emulsion Polymerization

12 g of Ludox TM-40 dispersion, 88 g of water, and 10 g of monomer (MMA or MMA/St mixture) were weighed into a 250 mL three-necked round-bottom glass flask equipped with a condenser. The pH of the dispersion was adjusted to around 5.5 by adding 0.1 M HCl (aq) to the liquid mixture unless otherwise stated. The flask was steadily stirred by a magnetic bar at 300 rpm, and oxygen was removed by nitrogen purging for 30 min. At the same time, the temperature was raised to 50–80 °C, and KPS solution (0.05 g in 1 g water) was injected to start the polymerization reaction. The polymerization reaction proceeded for at least 12 h to obtain the milky emulsion latexes. To perform the emulsion polymerization under different salt conditions, Ludox TM-40 silica sol was first dialyzed against pure water for 7 d to remove any free ions in the silica sol, and then different amounts of salts (NaCl or other salts) were added to adjust the specific ion concentration in the reaction system.

### Particle Size

The particle size and size distribution of latexes were determined using a dynamic light scattering particle size analyzer (ZetaPALS, Brookhaven, United States). A dispersion of 0.1 g of particles in 10 mL of water was prepared and diluted continuously with pure water to 0.05 wt %, and three cycles were performed to obtain the average particle size of latexes.

### Latex Morphology

Scanning electron microscopy was used to obtain the morphology of latexes (S-4800, Hitachi, Japan). A small amount of dispersion-containing latexes was diluted with ultrapure water to a concentration < 0.1 wt %, dropped onto a silicon wafer, and dried under ambient conditions. Gold was sputtered onto the sample to enhance its conductivity. The SEM image was conducted at 3 kV accelerating voltage.

### Interfacial Tension between Monomers and Water

The interfacial tension between monomers and water was measured through a pendant drop tensiometer (OCA15EC, Dataphysics Instruments GmbH, Germany). Water droplets were extruded into a monomer mixture (St and MMA) with different monomer ratios, and the interfacial tension between monomers and water was measured using capturing the shape of the pendant water drop, and the interfacial tension was calculated by the built-in software.

### Loading Rate of Silica Nanoparticles

The sample is centrifuged repeatedly at least three times to remove free silica nanoparticles before thermogravimetric analysis (TGA) measurement. The loading rate of silica nanoparticles can be calculated from the following equation if 100% monomer conversion can be assumed.
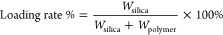


In a typical polymerization recipe (Table S1), the weight of silica nanoparticles is 12 g × 0.4 = 4.8 g. Therefore, the maximum loading rate of silica nanoparticles is 4.8/(4.8 + 10) × 100% = 32.4 wt %. The silica loading rate of hybrid latexes can be determined experimentally by TGA. TGA was performed by a Mettler-Toledo TGA instrument to analyze and determine the complete decomposition temperature of hybrid latexes. The hybrid latexes were dried to a constant weight under vacuum at ambient conditions before measurement. A sample weighing between 5 and 10 mg was placed in a crucible and then subjected to TGA under programmed heating. A nitrogen gas sweeping atmosphere was used to avoid oxidation, and the flow rate was 50 mL/min. The temperature range was from 50 to 600 °C with a heating rate of 10 °C/min.

## Results and Discussion

### Temperature

According to the possible nucleation mechanism for Pickering emulsion polymerization proposed by Bon and Lotierzo, the precipitation of low-molecular-weight radicals on the surface of silica nanoparticles is identified as a crucial step during nucleation.^28^ Consequently, factors influencing the precipitation of silica nanoparticles on the latex surface during Pickering emulsion polymerization will inevitably affect the morphology of the latexes and the loading rate of the silica nanoparticles. In practical emulsion polymerizations, the reaction temperature is usually in the range of 70–90 °C to accelerate the initiator decomposition rate and polymerization rate. The interfacial tension between oil and water is reduced at an elevated reaction temperature, thereby affecting the adsorption of silica nanoparticles on the surface of latexes. To investigate the influence of reaction temperature on the morphology of PMMA/SiO_2_ latexes, Pickering emulsion polymerization was conducted at different temperatures from 50 to 80 °C (Table S1).

**Figure 1:**
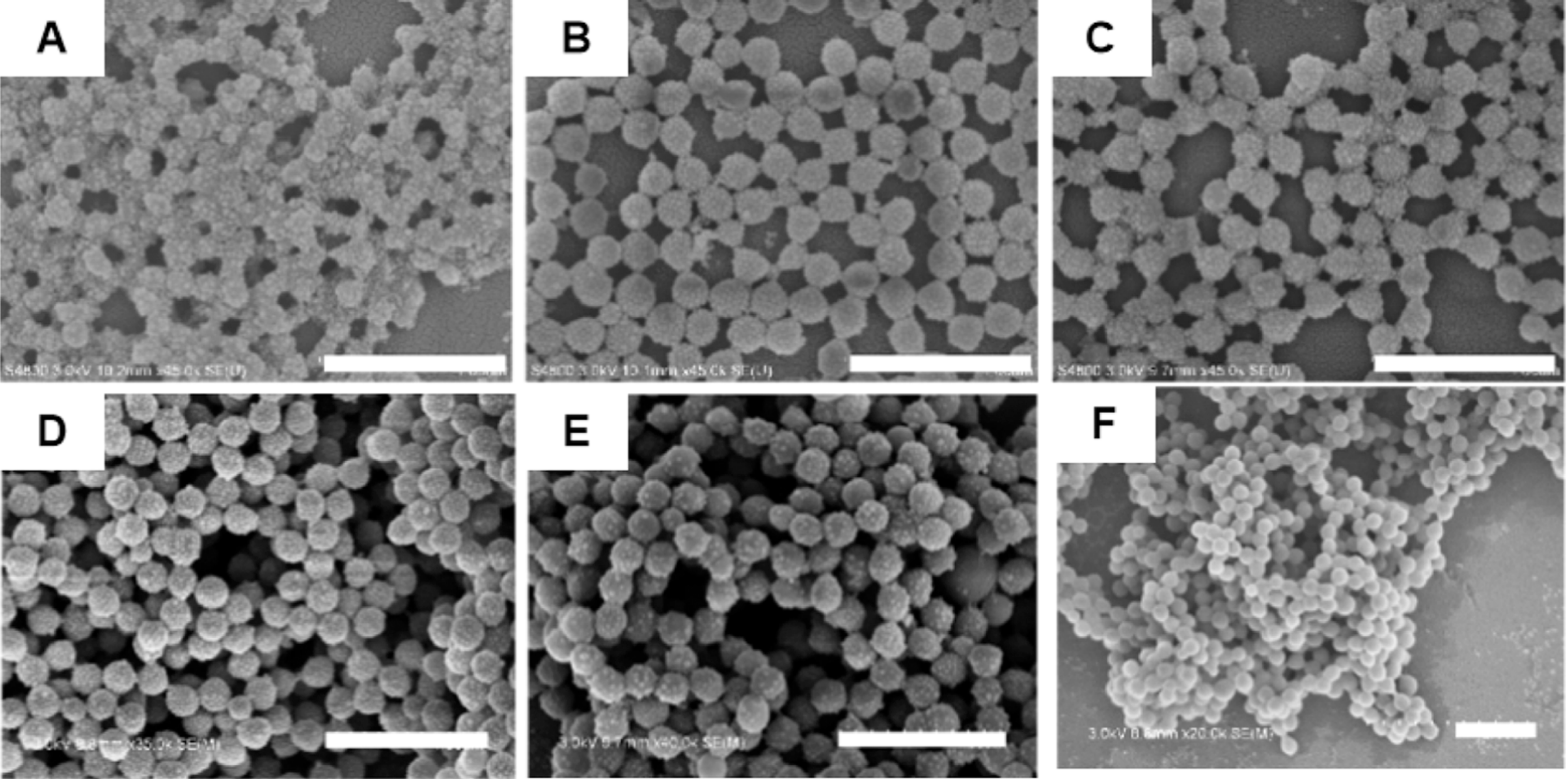
SEM images of PMMA/SiO_2_ latexes prepared at different temperatures: (A) 50; (B) 55; (C) 60; (D) 65; (E) 70; and (F) 80 °C. Scale bar is 1 μm for all images.

**Figure 2:**
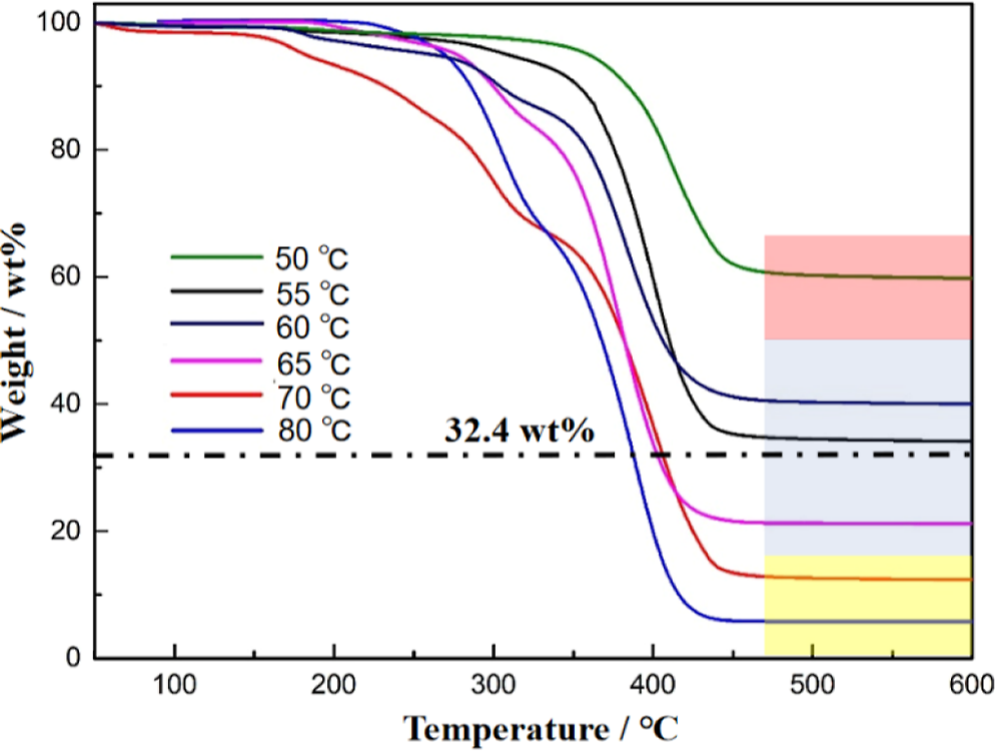
TGA profiles of PMMA/SiO_2_ hybrid latexes prepared at different reaction temperatures (50–80 °C). The dashed line (32.4 wt %) indicates the maximum loading rate of silica nanoparticles onto PMMA latexes (see Materials and Methods for details).

It can be observed from SEM images that with the gradual increase in reaction temperature, the surface density of silica nanoparticles on PMMA particles gradually decreases (Figure 1A–F). At low emulsion polymerization temperature (50–60 °C), the surface of PMMA latexes is fully covered by SiO_2_ nanoparticles (Figure 1A–C). At emulsion polymerization of 65 and 70 °C, the surface of PMMA latexes is only partially covered by silica particles (Figure 1D,E), and as the temperature further increases, the surface of PMMA latexes becomes smooth, with no observable adsorption of silica nanoparticles (Figure 1F). The reason for this experimental phenomenon is that the increase in reaction temperature reduces the interfacial tension between monomers and water. The decrease in interfacial tension reduces the detachment energy of inorganic particles from the interface, making it easier for them to detach.^3^ Therefore, the increase in the reaction temperature results in a reduction in the adsorption of silica nanoparticles on the surface of hybrid latexes. To quantitatively investigate the reaction temperature on the amount of silica particles adsorbed on PMMA latexes in Pickering emulsion polymerization, TGA was performed to measure the loading rate of silica particles on hybrid latexes calcined at 500 °C. From the TGA profiles, it can be observed that the residual mass ratio of hybrid latexes at 500 °C gradually decreases with the increase in the reaction temperature, indicating a reduction in the adsorption of silica on the surface of PMMA latexes. If a 100% monomer conversion can be assumed after 24 h of emulsion polymerization, the maximum 32.4 wt % loading rate of silica nanoparticles can be expected if all the silica nanoparticles are adsorbed onto PMMA latexes (see Materials and Methods for details). At a high polymerization temperature of 70–80 °C (purple region, Figure 2), the mass ratio of silica nanoparticles is below 15 wt % of the total weight of hybrid PMMA/SiO_2_ latexes. This indicates that most of the silica nanoparticles are not adsorbed onto PMMA latexes, and a large proportion of silica nanoparticles exist as free particles at the end of emulsion polymerization. When the reaction temperature is decreased to the range of 55–65 °C, the loading rate of silica nanoparticles is between 20 and 50 wt % (blue region, Figure 2). The loading rate of silica nanoparticles is gradually increased above 20 wt % and reaches around 32.4 wt % with a reaction temperature of 60 and 65 °C. This means that the silica nanoparticles approach full adsorption during emulsion polymerization, and no free silica particles exist at the end of emulsion polymerization. It is observed that the loading rate of silica nanoparticles is higher than 32.4 wt %, maximum loading rate of silica nanoparticles, when the reaction temperature is lowered to 55 °C (black curve, Figure 2). As discussed in the previous paragraph based on observation from SEM images (Figure 1), the lowered reaction temperature should promote silica particle adsorption onto the PMMA latex surface based on interfacial energy considerations. However, the promotion of silica nanoparticles onto PMMA latexes should not lead to a loading rate higher than 32.4 wt % if full conversion is assumed. The abnormal phenomena can be attributed to the fact that the decomposition of the radical initiator and polymerization rate are greatly reduced at the temperature below 60 °C, and the conversion of monomers is not complete even after 24 h of emulsion polymerization. First, the unpolymerized monomers will evaporate before TGA measurement; second, the presence of unpolymerized monomers in PMMA/SiO_2_ hybrid latexes will decrease the apparent glass-transition temperature of the latexes, which will render the latexes sticky at room temperature. Therefore, separation of the latexes from the as-prepared dispersion by centrifugation may have brought excess free silica nanoparticles into the PMMA/SiO_2_ hybrid latex sediments due to the sticky property of the latexes.

### Monomer Type

The monomers with different hydrophobicities can result in varying hydrophobicities of the generated oligomer radicals and swollen latexes. Therefore, the interfacial tension between monomers/swollen and water is also different, affecting the precipitation of oligomer radicals on silica nanoparticles. In the previous reports, raspberry-like hybrid latexes are obtained when MMA is employed as a monomer with unmodified silica nanoparticles, while the silica nanoparticles do not adsorb onto latexes when styrene is used as a monomer.^18^ To incorporate styrene monomer into Pickering emulsion polymerization, several strategies such as addition of hydrophilic monomers and glycerol-functionalized silica sol are employed to enhance the loading rate of silica nanoparticles onto polystyrene latexes.^27,28^ The influence of monomer type on the adsorption of silica particles onto the polymer latexes needs systematic investigation. When pure styrene was used as a monomer in Pickering emulsion polymerization (Table S2), silica nanoparticles cannot adsorb onto PSt latexes from high to low pH values (Figure S1A–C). In contrast, when MMA was used as the monomer, well-defined PMMA/SiO_2_ hybrid latexes were obtained at around pH 5.5, which is consistent with a previous report. To investigate the underlying mechanism of this phenomenon, styrene and MMA of different ratios are used as a monomer mixture in Pickering emulsion polymerization to investigate the loading rare of silica nanoparticles on polymer latexes (Table S3).

**Figure 3:**
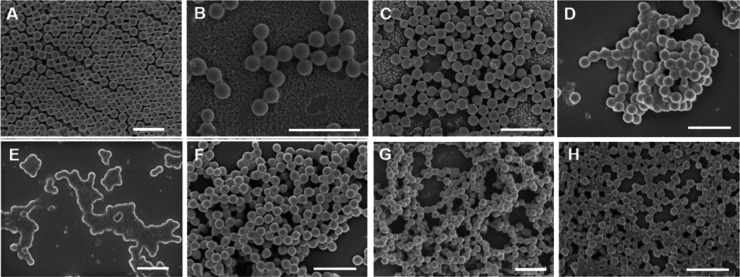
SEM images of PSt-PMMA/SiO_2_ hybrid particles obtained from Pickering emulsion polymerization of styrene and MMA in different mass ratios. (A) 60:40; (B) 50:50; (C) 40:60; (D) 30:70; (E) 20:80; (F) 15:85; (G) 10:90; and (H) 5:95. Scale bar is 1 μm for all images.

It can be observed that when a St and MMA monomer mixture is used for Pickering emulsion polymerization to prepare hybrid latexes, hybrid latexes cannot be observed under most experimental conditions when the mass ratio of St is over 15 wt % (Figure 3A–F). Only when the mass ratio of St/MMA is reduced to below 10:90 can latexes with silica nanoparticles adsorbed on the surface of polymer latexes be observed (Figure 3G,H). The above experimental results indicate that PMMA/PS/SiO_2_ hybrid latexes can only be prepared when the mass fraction of MMA in the monomers is ≥90 wt %. To further explore the loading rate of silica nanoparticles on latexes, experiments were conducted with a St mass ratio within 10 wt % in the monomer mixtures (Table S4).

**Figure 4:**
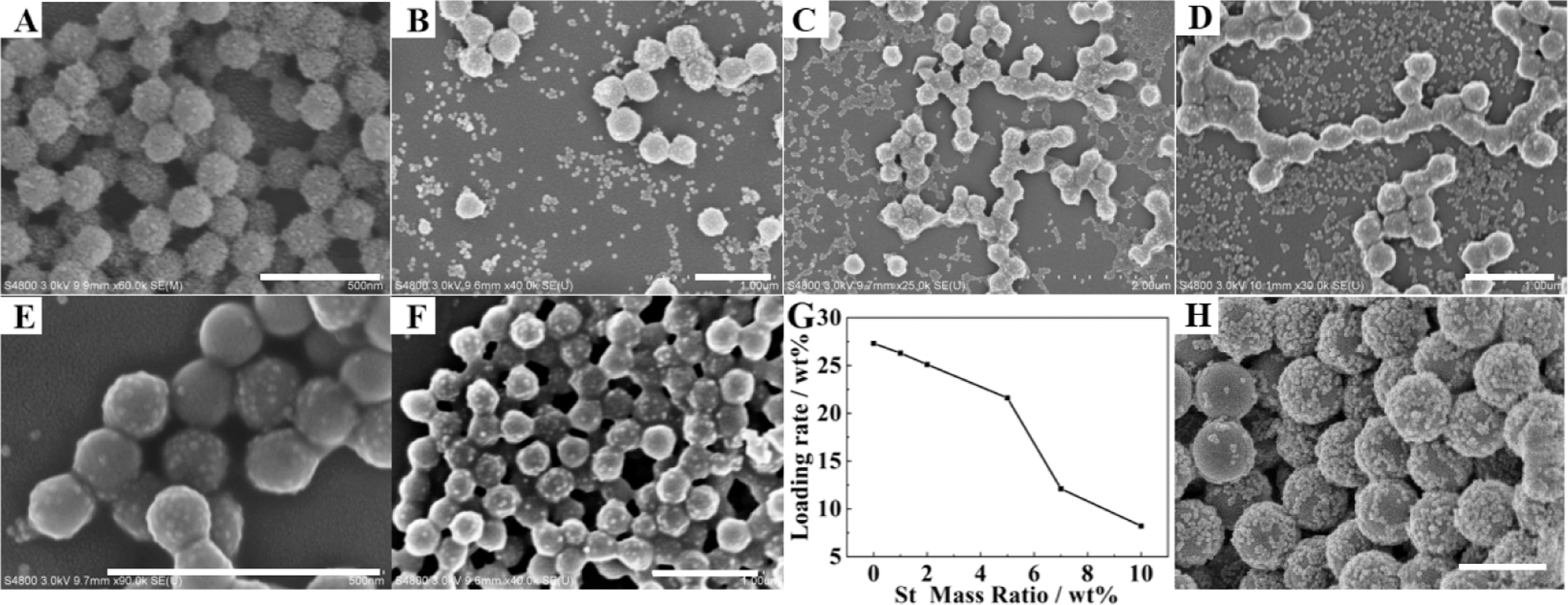
SEM images of latexes obtained by Pickering emulsion polymerization of St/MMA monomer mixtures with increasing St mass ratio within 10 wt %. (A) 0; (B) 1.0; (C) 2.0; (D) 5.0; (E) 7.0; and (F) 10 wt %. Scale bar is 1 μm for all images. (G) Relationship between the St monomer mass ratio and silica nanoparticle loading rate and (H) SEM image of latexes obtained by Pickering emulsion polymerization using pure St monomer with 5.0 wt % ethanol in water.

In the absence of St monomer, the PMMA latex surface is nearly fully covered with silica nanoparticles, and almost no free silica nanoparticles are observed in the background (Figure 4A). With increasing mass ratio of St from 1.0 to 5.0 wt % in the monomer mixture, the surface coverage of silica nanoparticles is obviously reduced as observed from SEM images, and free silica nanoparticles can be clearly seen in the background (Figure 4B–D). With St mass ratio above 5.0 wt %, the silica nanoparticles are only partially covered or scattered on latexes (Figure 4E,F). With increasing St mass ratio in the monomer mixture from 0 to 10.0 wt %, the loading rate of silica nanoparticles in PMMA/SiO_2_ latexes decreases from 30 wt % to below 10 wt %, as determined from TGA profiles (Figure 4G). It is known that the interfacial tension between the monomer mixture and water is increased with increasing St mass ratio in the monomer mixture because St has a higher interfacial tension with water (31.6 mN/m) than that of MMA (13.3 mN/m). As reported, monomers with high hydrophilicity can be better wetted by unmodified inorganic nanoparticles, facilitating the adsorption of silica nanoparticles on the surface of polymer latexes, forming hybrid latexes.^28^ The solubility of MMA (15.9 g/L) in water is much higher than that of styrene in water (0.31 g/L), which makes MMA a more hydrophilic monomer, and this also leads to reduced interfacial tension between water and the MMA monomers when MMA is saturated in the aqueous phase at the reaction temperature.^31^ Hence, in cases where there is enough MMA in the monomer mixtures, the loading rate of silica nanoparticles on latexes is high, and MMA, the primary radical, as well as its oligomers will have better affinity toward unmodified silica nanoparticles. Another possible reason is that as the interfacial tension between the monomers and water decreases with more MMA, making it more favorable for the low-molecular-weight radicals to adsorb on the surface of silica nanoparticles.^32^ If the above argument is true, the increase in St solubility and reduction of interfacial tension with the aqueous phase should promote the adsorption of silica nanoparticles onto latexes. To verify this hypothesis, ethanol, being both miscible with water and St, is added into the emulsion polymerization as a second solvent to increase the solubility of St in the aqueous phase and to reduce the interfacial tension between St and aqueous phase (Table S5). It is revealed that silica nanoparticles are adsorbed onto a pure PSt latex surface with only ∼5 wt % addition of ethanol into the emulsion polymerization (Figure 4H). This result further verifies the above proposed mechanism that monomer solubility in water is a crucial factor in promoting the adsorption of silica nanoparticles onto polymer latexes in emulsion polymerization. There are tens of monomers with varying solubilities currently employed in emulsion polymerization for various purposes,^30^ and the above results indicate that Pickering emulsion polymerization in the presence of unmodified silica nanoparticles is restricted to a very limited scope of monomers of suitable solubilities in water in the absence of a second solvent. Therefore, more research endeavors on the solution properties such as electrolyte concentrations and types are needed to clarify the underlying mechanism of silica particle adsorption onto polymer latexes in emulsion polymerization.

### Electrolytes

**Figure 5:**
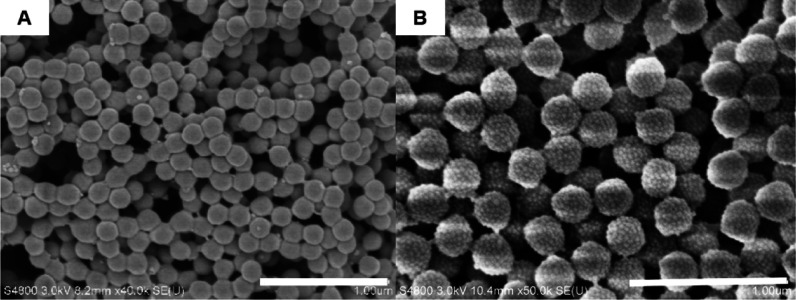
SEM images of PMMA latexes obtained by Pickering emulsion polymerization in the presence of silica nanoparticles (Ludox TM-40). (A) Silica sol is dialyzed against water for 7 d and (B) silica sol is used as received. Scale bar is 1 μm for both SEM images.

The presence of salts in an aqueous solution is one of the most influential factors that can change the physicochemical properties of a solution, and electrolytes should play critical roles in determining the final location of silica nanoparticles as free particles or adsorbed particles on polymer latexes in emulsion polymerization.^33^ It is revealed that salt concentration in the emulsion polymerization in the presence of nanogels can produce patchy or Janus colloids, and the surface coverage of nanogels on PSt colloids can be increased with low concentrations (≤10 mM) of NaCl, which is used to effectively suppress the electrical double layer of the nanogels.^34^ The question quickly arises that in Pickering emulsion polymerization in the presence of unmodified silica nanoparticles as a stabilizer, what is the role of electrolytes in determining the loading efficiency of silica nanoparticles onto a latex surface? It is well known that Ludox silica nanoparticles contain free salts in the original dispersion because the preparation process involves sodium silicates as precursors,^35,36^ and it is reported that a sodium ion can serve as a stabilizing cation for the silica nanoparticles.^37^ To elucidate the potential role of free salts intrinsic in the Ludox TM-40 silica sol on silica nanoparticle adsorption on polymer latexes, the Ludox TM-40 silica sol was first dialyzed against pure water in a dialysis bag for 7 days with clean water replacement each day to remove all free and excess ions. Subsequently, both pristine and dialyzed Ludox TM-40 silica sol was used for Pickering emulsion polymerization with MMA as the monomer under suitable reaction conditions to observe the silica nanoparticle adsorption on PMMA latexes (Table S6). With dialyzed silica sol, surprisingly only PMMA latexes with bare and smooth surfaces are obtained, and almost no silica particles are adsorbed onto the surface of PMMA latexes (Figure 5A). This is in distinctive contrast to hybrid PMMA/SiO_2_ latexes prepared in the presence of pristine silica nanoparticles (Figure 5B). This preliminary result inspires us to further investigate the electrolyte effects on the loading rate of silica nanoparticles on the surface of PMMA latexes with varying amount of NaCl addition during emulsion polymerization (Table S7).

**Figure 6:**
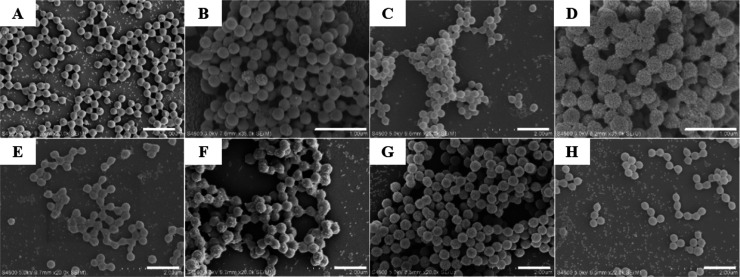
SEM images of PMMA/SiO_2_ latexes prepared by emulsion polymerization in the presence of dialyzed silica nanoparticles with increasing NaCl concentration in water. (A) 0.5; (B) 1; (C) 4; (D) 5; (E) 6; (F) 8; (G) 10; and (H) 20 mM.

**Figure 7:**
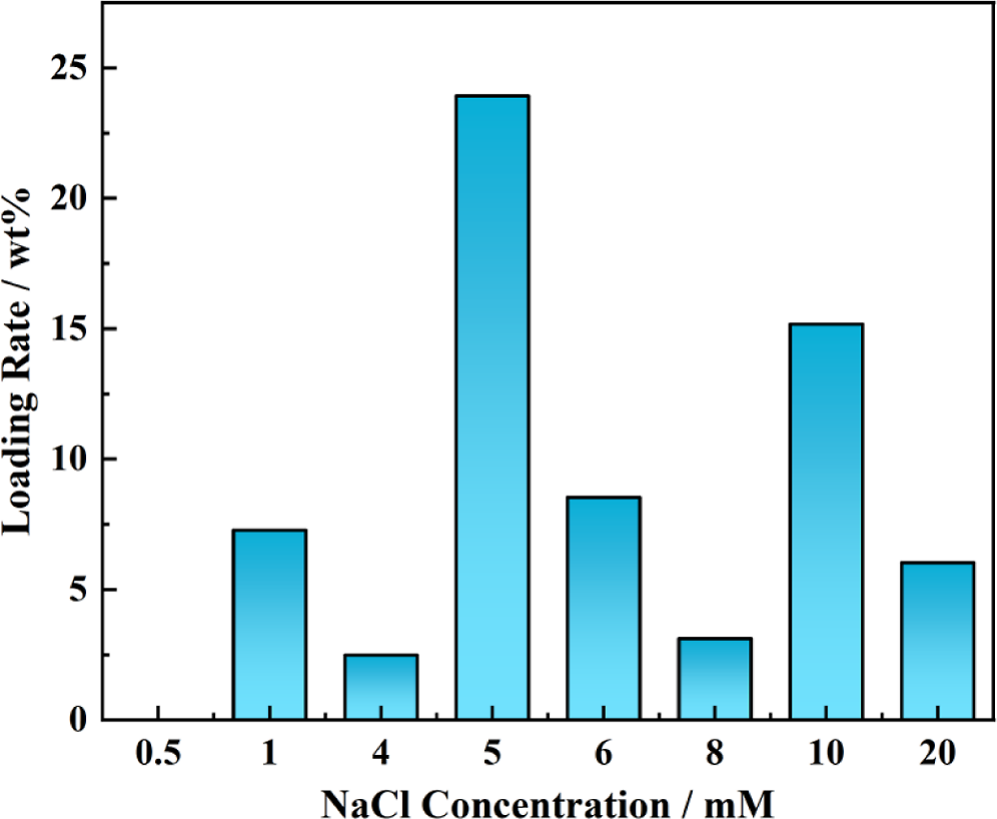
Loading rate of silica nanoparticles in PMMA/SiO_2_ hybrid latexes determined from TGA measurements. The PMMA latexes are prepared by emulsion polymerization in the presence of dialyzed silica nanoparticles with increasing NaCl concentration.

It is evident that the addition of NaCl can result in the adsorption of silica particles on the latex surface (Figure 6). At low NaCl concentrations of 0.5 mM to below 5 mM, the PMMA latex particle surface is basically bare without any adsorption of silica nanoparticles (Figure 6A–C). When the NaCl concentration is increased to 5 mM, a dense accumulation of silica particles is observed on the surface of PMMA latexes, reaching maximum adsorption (Figure 6D). However, as the NaCl concentration continues to increase in the range of 6–10 mM, partial coverage of silica particles on the latex particle surface is observed, and the surface coverage is decreased compared to that of latexes prepared with added 5 mM NaCl in the aqueous phase (Figure 6E–G). When the NaCl concentration is increased to 20 mM, only latexes with bare surface are obtained, and basically no silica particles are adsorbed on the PMMA latex surface, possibly due to the excessive screening of an electrical double layer of silica nanoparticles at high salt concentration, preventing their adsorption on the latex surface (Figure 6H). As determined by TGA measurements, the loading rate of silica nanoparticles in PMMA latexes reaches the highest value (∼23.9 wt %) at a NaCl concentration of 5 mM (Figure 7). The loading rate of silica nanoparticles in PMMA latexes is decreasing with lower or higher concentrations of NaCl than 5 mM. At lower NaCl concentrations (<5 mM), the silica nanoparticles may not be sufficiently stabilized by the counterion (sodium ions), and at higher concentrations, the electrical double layer of silica nanoparticles is overly suppressed to induce aggregation of silica nanoparticles.^38^ These results demonstrate that the presence of a suitable concentration of salt in emulsion polymerization is crucial to promote the adsorption of silica nanoparticle onto latex surfaces. In the original report of Bon et al., high loading rate of silica nanoparticles in PMMA latexes can only be achieved in the pH range of 5–6, preferably with pH 5.5. This incites us to wonder whether the adsorption of silica nanoparticles can be promoted with the assistance of a suitable amount of NaCl (5 mM) during emulsion polymerization at high pH ranges (Table S8). Unfortunately, the outcome is not as expected that nearly all silica nanoparticles are not adsorbed onto the PMMA latexes when the pH is elevated to 7–9 (Figure S2A–C) and only when the pH is lowered to 6 can surface coverage of silica nanoparticles be observed from SEM images (Figure S2D). At pH value above 7.0, the surface of silica nanoparticles will be highly negatively charged as the surface silanol group will be mostly deprotonated.^18^ This is why a cationic initiator, 2,2′-azobis[2-methylpropionamidine] dihydrochloride (AIBA) instead of potassium persulfate (KPS), was preferentially chosen to promote the adsorption of silica nanoparticles onto the latex surface in previous research studies.^27^ In the absence of electrostatic attraction when KPS is used as an initiator, the underlying mechanism of adsorption of silica nanoparticles onto PMMA latexes still cannot be well explained based on the above results.

**Figure 8:**
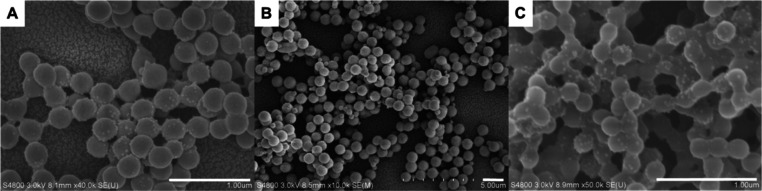
SEM images of PMMA/SiO_2_ latexes prepared by emulsion polymerization in the presence of dialyzed silica nanoparticles with different salts. (A) KCl (5 mM); (B) CaCl_2_ (5 mM); and (C) Na_2_SO_4_ (2.5 mM). Scale bar is 1 μm for all images.

To further clarify the critical role of electrolytes in promoting the adsorption of silica nanoparticles onto polymer latexes in Pickering emulsion polymerization, potassium chloride (KCl), calcium chloride (CaCl_2_), and sodium sulfate (Na_2_SO_4_) are subsequently introduced into Pickering emulsion polymerization in the presence of dialyzed silica nanoparticles (Table S9). With KCl and CaCl_2_ as added electrolytes, no silica nanoparticles can adsorb onto latex particle surfaces, and only latexes with smooth surfaces are observed (Figure 8A,B), while the adsorption of silica nanoparticle onto PMMA latexes is clearly observed when Na_2_SO_4_ is added in emulsion polymerization (Figure 8C). This leads us to realize the critical and specific role of the sodium ion (Na^+^). Pristine Ludox silica nanoparticles cannot get effectively stabilized with other cations such as K^+^ and Ca^2+^, and subsequently, during Pickering emulsion polymerization, the silica nanoparticles cannot get adsorbed onto latexes. With K^+^ or Ca^2+^ other than Na^+^ in the silica sol, the cations can get well dispersed in water, but they actually do not act as stabilizers for the silica nanoparticles as Na^+^ cations do due to size or charge differences.^39^ Therefore, these dialyzed silica nanoparticles without the presence of Na^+^ cations will not adsorb on the PMMA latexes in the emulsion polymerization. The above results further demonstrate that the cation type is crucial to the loading rate of silica nanoparticles onto polymer latexes which is first revealed in this study.

## Conclusions

To conclude, emulsion polymerization in the presence of unmodified silica nanoparticles without the use of an auxiliary comonomer and a cationic initiator is systematically explored to reveal the influence of various factors on the loading rate of silica nanoparticles onto PMMA latexes. It reveals that the emulsion polymerization temperature, monomer solubility, salt concentration, and cation type can all play a role in determining the adsorption of silica nanoparticles onto polymer latexes in emulsion polymerization. The emulsion polymerization temperature is primarily related to changes in the interfacial tension between the monomer and water. At higher temperatures, the interfacial tension between water and monomer as well as monomer swollen latexes is reduced, leading to easier detachment of silica nanoparticles from the interface, and therefore, silica nanoparticles are less favorable for adsorption on the surface of hybrid latexes. With an increase of hydrophobic monomer such as St in the monomer mixture, the loading rate of silica nanoparticles is gradually reduced because of the solubility of St in water being much lower than that of PMMA. The concentration of NaCl can significantly influence the loading rate of silica nanoparticles on PMMA latexes when dialyzed silica nanoparticles are used as stabilizers. By thoroughly dialyzing the silica sol against pure water, the silica nanoparticles can hardly adsorb onto the surface of latexes under the whole pH range. The highest silica loading efficiency reaches 23.9 wt % at a NaCl concentration of 5 mM. It is proved that the presence of Na^+^ cations is crucial to the adsorption of silica nanoparticles onto PMMA latexes as addition of KCl or CaCl_2_ cannot lead to hybrid PMMA/SiO_2_ latexes under favorable pH conditions. Soap-free polymer dispersion is highly desired in a variety of industries, and Pickering emulsion polymerization that employs commercial monomers in the presence of inorganic nanoparticles offers a promising approach toward this goal. This study provides valuable insights into experimental factors influencing the loading rate of silica nanoparticles and can serve as a benchmark for further research on Pickering emulsion polymerization mechanisms and development of high-performance polymer dispersions based on acrylate monomers, which holds significance in coating, adhesive, and ink industries.

## Supporting Information Available

Pickering emulsion recipes and SEM images of polymer latexes (PDF)
