# Supplementary material for: Unraveling the Complexities of Silica Nanoparticle Adsorption onto Polymer Latexes in Pickering Emulsion Polymerization
Source: Langmuir. 2024 Aug 19;40(35):18652–60. doi: 10.1021/acs.langmuir.4c02219 (PMC11375769; doi:10.1021/acs.langmuir.4c02219)
Supplement: Supplementary file 1 — la4c02219_si_001.pdf [file la4c02219_si_001.pdf]

Supporting Information for:

Unraveling the Complexities of Silica Nanoparticles Adsorption onto Polymer Latexes in Pickering Emulsion Polymerization

Zekai Shen[1], Tianheng Wang[1], Jing Luo[1], Ren Liu [1], To Ngai[1,2] Guanqing Sun[1*],*

1 Key Laboratory of Synthetic and Biological Colloids, Ministry of Education, School of Chemical and Material Engineering, Jiangnan University, Wuxi, Jiangsu, 214122, PR China

2. Department of Chemistry, The Chinese University of Hong Kong, Shatin N.T., 999077, Hong Kong SAR, China.

To whom correspondence should be addressed: tongai@cuhk.edu.hk & guanqingsun@jiangnan.edu.cn

Table S1. Pickering emulsion polymerization recipes with increasing polymerization temperature from 50 ℃ - 80 ℃.

Entry No.	Silica sol/g	MMA/g	Water/g	KPS/g	pH	Temp./℃
1	12	10	88	0.05	5.68	50
2	12	10	88	0.05	5.63	55
3	12	10	88	0.05	5.56	60
4	12	10	88	0.05	5.52	65
5	12	10	88	0.05	5.32	70
7	12	10	88	0.05	5.28	80

Table S2. Pickering emulsion polymerization recipes with St as the monomer from pH 4 - 7.

Entry No.	Slica sol/g	St/g	Water/g	KPS/g	Temp./℃	pH
1	12	10	88	0.05	65	7.02
2	12	10	88	0.05	65	5.89
3	12	10	88	0.05	65	4.10

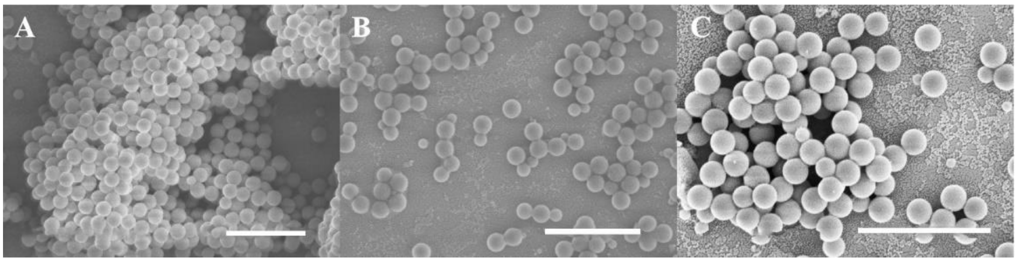

Figure S1. SEM images of PSt latexes prepared by Pickering emulsion polymerization from pH range 4 – 7(Table S2). **A**. pH=7.02; **B**. pH=5.89; **C**. pH= 4.10. Scale bar is 2 μm for all images.

Table S3. Pickering emulsion polymerization with different St/MMA mass ratios.

Entry No.	Silica sol/g	MMA/g	St/g	Water/g	KPS/g	pH	Temp./℃

1	12.0	4.0	6.0	88.0	0.05	5.61	65
2	12.0	5.0	5.0	88.0	0.05	5.53	65
3	12.0	6.0	4.0	88.0	0.05	5.53	65
4	12.0	7.0	3.0	88.0	0.05	5.46	65
5	12.0	8.0	2.0	88.0	0.05	5.57	65
6	12.0	8.5	1.5	88.0	0.05	5.59	65
7	12.0	9.0	1.0	88.0	0.05	5.43	65
8	12.0	9.5	0.5	88.0	0.05	5.61	65

Table S4. Pickering emulsion polymerization with St mass ratios in the monomer mixture within 10 wt%.

Entry No.	Silica sol/g	St/g	MMA/g	Water/g	KPS/g	pH	Temp./℃
1	12.0	0.0	10.0	88.0	0.05	5.56	65
2	12.0	0.1	9.9	88.0	0.05	5.52	65
3	12.0	0.2	9.8	88.0	0.05	5.50	65
4	12.0	0.5	9.5	88.0	0.05	5.52	65
5	12.0	0.7	9.3	88.0	0.05	5.47	65
6	12.0	1.0	9.0	88.0	0.05	5.59	65

Table S5. Pickering emulsion polymerization of pure St in the presence of 5.0 wt% ethanol in the reaction system.

Silica sol/g	Water/g	St/g	EtOH/g	KPS/g	pH	Temp./℃
12.0	88.0	10.0	5.0	0.05	5.26	65

Table S6. Pickering emulsion polymerization with pristine and dialyzed silica sols.

Entry No.	Silica sol/g	MMA/g	Water/g	KPS/g	pH	Temp./℃
1	12 (dialyzed)	10	88	0.05	5.50	65
2	12 (pristine)	10	88	0.05	5.45	65

Table S7. Pickering emulsion polymerization in the presence of dialyzed silica nanoparticles with increasing salt concentration.

Entry No.	Silica sol (dialyzed)/g	MMA/g	Water/g	[NaCl]/mM	KPS/g	Temp./℃
1	12.0	10.0	88.0	0.5	0.05	65
2	12.0	10.0	88.0	1.0	0.05	65
3	12.0	10.0	88.0	4.0	0.05	65
4	12.0	10.0	88.0	5.0	0.05	65
5	12.0	10.0	88.0	6.0	0.05	65
6	12.0	10.0	88.0	8.0	0.05	65
7	12.0	10.0	88.0	10.0	0.05	65
8	12.0	10.0	88.0	20.0	0.05	65

Table S8. Pickering emulsion polymerization with Pickering emulsion polymerization in the presence of dialyzed silica nanoparticles with 5 mM NaCl in the pH range 6 – 9.

Entry No.	Silica sol (dialyzed)/g	MMA/g	Water/g	[NaCl]/mM	KPS/g	Temp./℃	pH
1	12	10	88	5	0.05	65	9.04
2	12	10	88	5	0.05	65	8.09
3	12	10	88	5	0.05	65	6.96
4	12	10	88	5	0.05	65	6.06

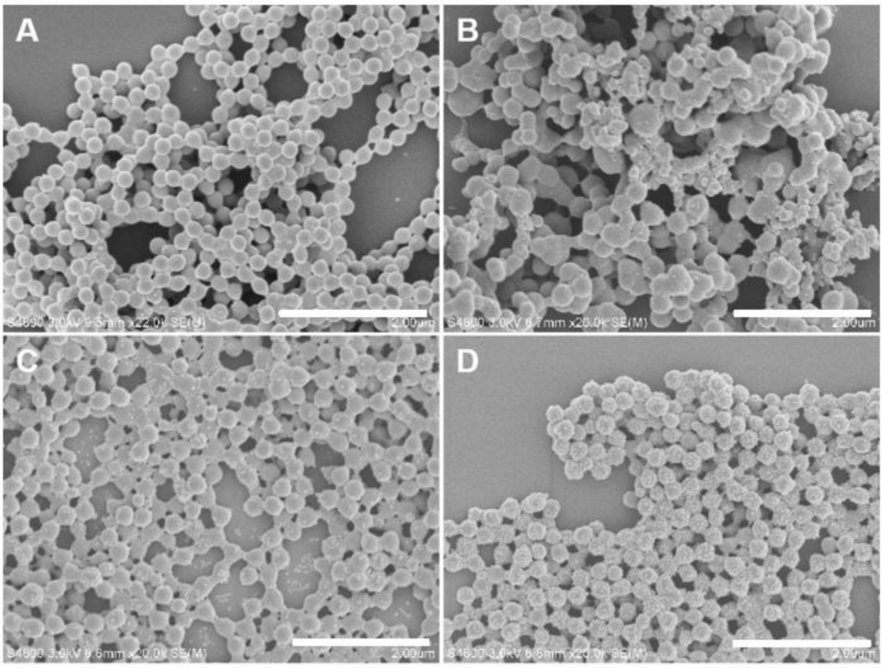

Figure S2. SEM images of latexes from Pickering emulsion polymerization in the presence of dialyzed silica nanoparticles with 5 mM NaCl in the pH range 6 – 9. **A**. pH=9; **B**. pH=8; **C**. pH=7; **D**. pH=6. Scale bar is 2 μm for all images.

Table S9. Pickering emulsion polymerization in the presence of dialyzed silica nanoparticles with added KCl, $CaCl_2$ and Na_2SO_4.

Entry No.	Silica sol (dialyzed)/g	MMA/g	Water/g	pH	KPS/g	Temp./℃	[KCl]/mM	[$CaCl_2$]/mM	[Na_2SO_4]/mM
1	12	10	88		0.05	65	1	0	0
2	12	10	88		0.05	65	5	0	0
3	12	10	88		0.05	65	10	0	0
4	12	10	88		0.05	65	15	0	0
5	12	10	88		0.05	65	20	0	0
6	12	10	88		0.05	65	0	1	0
7	12	10	88		0.05	65	0	2	0
8	12	10	88		0.05	65	0	5	0
9	12	10	88		0.05	65	0	0	1
10	12	10	88		0.05	65	0	0	2.5
11	12	10	88		0.05	65	0	0	5
